# Mean Motor Nerve Conduction Velocity of Right Ulnar Nerve among Physically Trained Adult Males in a Tertiary Care Centre: A Descriptive Cross-sectional Study

**DOI:** 10.31729/jnma.7217

**Published:** 2022-06-30

**Authors:** Rekha Limbu, Nirmala Limbu, Rita Khadka, Priza Subedi, Dilmaya Limbu

**Affiliations:** 1 Department of Basic and Clinical Physiology, B.P. Koirala Institute of Health Sciences, Buddha Road, Dharan, Nepal

**Keywords:** *Nepal*, *nerve conduction*, *ulnar nerve*

## Abstract

**Introduction:**

Neural adaptation to physical training allows a person to better coordinate the activation of all relevant muscles producing maximum force. Nerve conduction velocity measures the speed of impulse along the motor neuron and is strongly associated with muscle contraction time. This study aimed to find out the mean motor nerve conduction velocity of the right ulnar nerve among physically trained adult males in a tertiary care centre.

**Methods:**

This descriptive cross-sectional study was conducted in the Neurophysiology Laboratory of a tertiary care centre from 3^rd^ November, 2019 to 2^nd^ November, 2020. Thirty young adult males who were engaged in the physical training at a training centre for more than 3 months were studied after receiving ethical approval from the Institutional Review Committee (Reference number: 1578/019). Motor nerve conduction parameters of the right ulnar nerve were measured and data were entered in Microsoft Excel. Statistical analysis was done using the Statistical Packages for the Social Sciences version 25.0. Point estimate at 95% Confidence Interval was calculated along with frequency and percentages for binary data, and mean and standard deviation for continuous data.

**Results:**

Among 30 males studied, the mean motor nerve conduction velocity of the ulnar nerve was found to be 61.02±5.86 m/sec (58.92-63.11 at 95% Confidence Interval). The mean distal latency and amplitude of the muscle action potential were 2.33±0.53 ms and 8.08±1.17 mv respectively.

**Conclusions:**

Our study found that the mean nerve conduction velocity of the ulnar nerve was similar when compared to studies conducted in similar settings.

## INTRODUCTION

Physical training induces adaptive changes within the nervous system that allow trainees to better coordinate activation of all relevant muscles resulting in maximal force production.^1^ A rapid Nerve Conduction Velocity (NCV) is an indicator of a short refractory period which allows for greater impulse frequency, thereby elevating muscle activation levels.^2^ A number of studies have found higher NCV in trained than untrained ones.^3^,^4^ It has also been mentioned that NCV is greater in the dominant limb as compared to the non-dominant limb.^3,5^

Every year, thousands of young males from different regions of Nepal attempt to recruit into the British army. The selection process is physically demanding so most youths join private training centre that provides them with necessary training. However, the study on nerve conduction velocity among trained individuals has not yet been conducted in Nepal.

This study aimed to find out the mean nerve conduction velocity of the motor component of the right ulnar nerve among physically trained adult males in a tertiary care centre.

## METHODS

This descriptive cross-sectional study was conducted from 3^rd^ November, 2019 to 2^nd^ November, 2020 in the Neurophysiology Laboratory, Department of Basic and Clinical Physiology at B.P. Koirala Institute of Health Sciences, Nepal. The ethical approval was obtained from the Institutional Review Committee (Reference number: IRC/1578/019). The procedure was fully explained and informed written consent was given by all the subjects enrolled on the study. The study group consisted of 30 young males undergoing physical training at least for more than 3 months with ages ranging from 18 to 23 years. They were selected from the Ex British Army Training Centre of Dharan by convenience sampling. Subjects with major systemic illness, peripheral nerve injuries, muscular-skeletal injuries, lumbar disc herniation, and compression syndrome of both extremities were excluded from the study. The sample size was calculated using the following formula:


$$\begin{array}{ll} \text{n} & =\frac{{\text{Z}}^{2}\times \sigma^{2}}{{\text{e}}^{\text{2}}}\\ & =\frac{1.96^{2}\times 12.39^{2}}{5^{2}}\\ & =24 \end{array}$$

Where,

n = minimum required sample size
Z = 1.96 at 95% Confidence Interval (CI)
σ = standard deviation of mean motor nerve conduction velocity of right ulnar nerve, 12.39^6^
e = margin of error

Hence, the minimum required sample size was 24. However, a sample size of 30 was taken for the study. A detailed history and clinical examination were performed using standard proforma of all subjects involved in the study. Anthropometric and cardiorespiratory variables of the subjects were studied. Motor nerve conduction parameters of the ulnar nerve in the right hand were recorded using Digital Nihon Kohden (NM420S_H636, Japan) by belly-tendon montage. For each site of stimulation, distal latency, proximal latency, amplitude, and NCV of Compound Muscle Action Potentials (CMAPs) was recorded. The room temperature was maintained at 26±2°Celsius during the recording.

The Motor Nerve Conduction Velocity (MNCV) was calculated through the determination of the time the stimulus took to cover the distance between the Proximal Latency (PL) and Distal Latency (DL) stimulation sites; that is, the time of conduction. This time, expressed in milliseconds (ms), may be calculated by the following formula:^7^ Time of conduction= PL-DL.

Latency may be defined as the time between the application of the stimulus and the beginning of the deflection of the M response obtained in the two sites of stimulation; proximal and distal. In order to have the distance between the two sites of stimulation measured, the centre between the electrodes used for the application of the stimulus was marked, both on the proximal and distal stimulation sites, measuring hence in millimetres (mm) the distance between them.^7^ The MNCV (in meters per second, m/s) was calculated using the following formula:^7^ MNCV (m/s) = Distance between the two stimulation sites (mm)/Time of conduction between the two stimulation sites (ms).

Data were collected and entered in Microsoft Excel. Statistical analysis was done using the Statistical Packages for the Social Sciences version 25.0. Point estimate at 95% Confidence Interval was calculated along with frequency and percentages for binary data, and mean and standard deviation for continuous data.

## RESULTS

Among 30 males studied, the mean nerve conduction velocity of the right ulnar nerve was found to be 61.02±5.86 m/sec (58.92-63.11 at 95% Confidence Interval). The anthropometric and cardiorespiratory variables of the males are tabulated below (Table 1).

**Table 1:** Anthropometric and cardiorespiratory variables (n = 30).

Variables	Mean±SD
Age (years)	18.58±0.90
Weight (kilograms)	61.17±4.80
Height (centimetres)	165.67±6.03
Body mass index (kg/m^2^)	22.55±2.47
Upper limb length (centimetres)	73.29±3.71
Pulse rate (beats per minute)	68.17±3.35
Systolic blood pressure (mm per Hg)	114.83±5.08
Diastolic blood pressure (mm per Hg)	72.00±7.34
Respiratory rate (breaths per minute)	16.83±1.03

**Figure 1:**
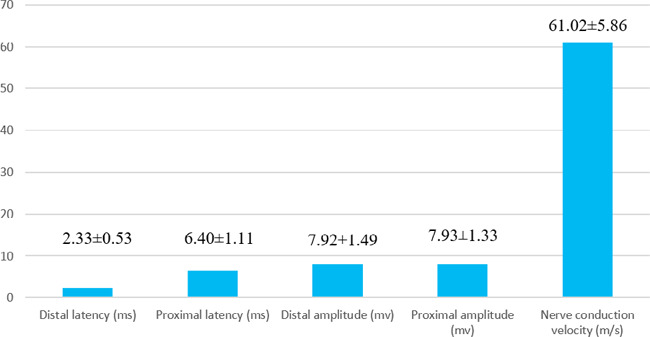
Motor conduction parameters of the ulnar nerve of trained males.

The mean values of distal and proximal latencies of the ulnar nerve were 2.33±0.53 ms and 6.40±1.11 ms respectively. Similarly, the mean values of distal and proximal amplitudes were 7.92±1.49 mv and 7.93±1.33 mv respectively (Figure 1).

## DISCUSSION

Our study aimed to electrophysiologically evaluate the ulnar nerve functions in young adult males undergoing physical training. The average duration of training was 4.25 ±1.06 months. In the present study, the mean nerve conduction velocity of the ulnar nerve was 61.02±5.86 m/sec.

A study done on the sprinters has reported the conduction velocity of the ulnar nerve lower than our result (47.95±12.39 m/s).^6^ However, the sample size was greater (n= 60) than our study. Another study has found the mean value of NCV in eight healthy pitchers relatively similar to our findings (64.40±7.34 m/s).^3^ Various studies have suggested the MNCV is higher in trained individuals. A significant increase in ulnar NCV following the strength training of immobilised thenar muscle has been reported.^8^ Similarly, higher NCV resulted after the single and multi-joint training.^9^ This might be related to neuromuscular adaptation responses induced by training.^10^

Nerve conduction velocity was found lower in the athletes frequently receiving endurance training (59.8±7.11 m/s) and higher in athletes receiving strength training, such as weight lifters (64.9 ±4.12 m/s).^10,11^ Studies have pointed out that the influence of physical activity is not the same for all types of exercise and that not all nerves may be affected in the same way.^5,12^ A number of studies have also mentioned that the functional overload may contribute to the increase of the diameter of the nerve fibres and the myelin sheath leading to higher NCV.^3,13^

This study can be further conducted on a larger sample size with more long duration of training. Based on the results-further studies will be conducted to determine the effect of pre- and post-training of similar subjects on both hands. However, interesting data were raised, despite the reduced number of the sample-which encourages us to carry on with the studies.

## CONCLUSIONS

In our study, the mean nerve conduction velocity of the right ulnar nerve was comparable to studies conducted in similar settings. The mean nerve conduction velocity could be different in dominant and non-dominant limbs.
